# Experience-Dependent Neuroplasticity in the Hippocampus of Bilingual Young Adults

**DOI:** 10.1523/ENEURO.0128-25.2025

**Published:** 2025-05-27

**Authors:** Federico Gallo, Toms Voits, Jason Rothman, Jubin Abutalebi, Yury Shtyrov, Andriy Myachykov

**Affiliations:** ^1^UiT The Arctic University of Norway, Tromsø N-9037, Norway; ^2^University of Gothenburg, Gothenburg 405 30, Sweden; ^3^Lancaster University, Lancaster LA1 4YW, United Kingdom; ^4^Nebrija Research Center in Cognition, Madrid 28043, Spain; ^5^Higher School of Economics, Moscow 109028, Russia; ^6^University Vita Salute San Raffaele, Milan 20132, Italy; ^7^Aarhus University, Aarhus 8000, Denmark; ^8^University of Macau, Taipa, Macau 999078, China

**Keywords:** bilingualism, expansion–renormalization, experience-dependent plasticity, hippocampus, MRI

## Abstract

Models of experience-dependent neuroplasticity predict that the acquisition and extensive use of a new skill trigger a nonlinear trajectory of neurostructural modifications, where initial expansion of relevant brain areas subsequently (once the skill is acquired) gives way to volumetric renormalization. Such predictions also apply in the domain of language during learning and/or simultaneous management of two (or more) linguistic systems. In a sample of 69 young adult Russian–English bilinguals, we tested the hypothesis that individual differences in bilingual engagement nonlinearly correlate with normalized volume of the hippocampus—a key learning-related brain region particularly amenable to experience-dependent plasticity. Results revealed an inverted U-shape association between second language engagement and left hippocampal gray matter volume. The present results replicate and expand the findings from aging populations, showing a nonlinear pattern of structural hippocampal plasticity in healthy young adults. These findings support the role of bilingualism as a promoter of experience-dependent neuroplasticity.

## Significance Statement

Bilingual experience has been associated with neurocognitive adaptations and linked to more favorable cognitive aging. The hippocampus, crucial in aging, has been previously shown to exhibit volumetric increases in response to language learning with some reports of nonlinear adaptations linked to bilingual experience. General models of neuroplasticity related to skill acquisition and bilingualism-specific models predict a morphological trajectory of volumetric expansions followed by renormalization of hippocampal volumes along the bilingual experience continuum. In this cross-sectional study we, for the first time, empirically tested this prediction in a sample of young individuals. In line with model predictions, our findings revealed an inverted U-shape relationship between second language engagement and left hippocampal volume, suggesting bilingualism as a source of experience-dependent neuroplasticity.

## Introduction

External influences such as the acquisition and sustained use of new skills can reshape the human brain through “experience-dependent plasticity” (Lindenberger and Lövdén, 2019). The relationship between structural changes and learning is not linear (Wenger et al., 2017a). Wenger and colleagues (Wenger et al., 2017b) showed a trajectory of gray matter (GM) expansion in the initial stages of motor skill learning, followed by a subsequent renormalization to pretraining volumes. Importantly, this inverted U-shape dynamic emerged in the presence of continuous practice “and” improving task performance. This and other similar results led to the formulation (Lövdén et al., 2013) and refinement (Lindenberger and Lövdén, 2019) of the “exploration–selection–refinement (ESR)_model” of experience-dependent plasticity, positing a trajectory of expansion and renormalization of brain structure associated with learning. Initially, the brain explores combinations of available neuronal microcircuits (Yuste et al., 2024) that can perform the relevant task. Increased coordinated activity results in neurostructural modifications via dendritic branching/synaptogenesis and/or the generation/modification of astrocytes and microglia (Zatorre et al., 2012). Subsequently, dopamine-mediated processes contribute to the selection and stabilization of the most efficient circuits (Dolan and Dayan, 2013). Finally, any excess synaptic connections are eliminated via efficiency-based mechanisms such as synaptic pruning.

“Bi-/multilingualism” is a known source of neurocognitive adaptations (Bialystok et al., 2012), affecting both language-related and domain-general brain structure/function and associated cognitive abilities (Grundy et al., 2017). Prior to the last decade, relevant work in bilingualism heavily relied on between-group comparisons of bilinguals and monolinguals. However, the field is presently transitioning to a more nuanced and ecologically valid approach, operationalizing bilingual experience as a continuum and shifting to within-group analyses (Rothman et al., 2023). This has promoted the development of theoretical models delineating the time-course of neurocognitive modifications expected to emerge at different stages of the experience.

Drawing from the ESR model, the “dynamic restructuring model” (DRM; Pliatsikas, 2020) applies its framework to detail how the trajectory of bilingualism-induced neuroplastic changes is expected to unfold over time under sustained engagement from L2 learning to maintenance/use. The DRM predicts a trajectory of expansion and subsequent renormalization across several brain regions with increasing L2 competence. One such brain structure is the hippocampus, implicated in aging, memory, and other domains of cognition. Research confirms dual language engagement (learning or use) is linked to augmented hippocampal volume across different age groups with mixed findings regarding the laterality of the effect. Bilingualism-related increases in hippocampal volume have been reported in the right (Mårtensson et al., 2012; Bellander et al., 2016; DeLuca et al., 2019) and left hemisphere (Li et al., 2017) or bilaterally (Voits et al., 2022, 2024; Coulter et al., 2024). As some of the studies cited above show bilingualism-related hippocampal increases, without revealing any subsequent decreases with increased L2 expertise, the predictions of DRM for the hippocampus are at least partly supported by empirical data. However, the existing literature mostly employs linear modeling and thus is unable to directly test the predictions of DRM. The only study using nonlinear modeling for the hippocampus in MCI patients (Voits et al., 2024) indeed finds an inverted U-shape relationship between hippocampal volumes and L2 engagement. Thus, the underlying data may be nonlinear, but linear effects can still be observed. [It is also true that these studies vary in terms of what regressors they employ in their analyses and/or show as explanatory, e.g., age of acquisition (AoA), proficiency, etc. This alone complicates a meaningful comparison of the studies or anything that can clearly be drawn from them in their aggregate. Being that they are so few to begin with, it is not clear that when some show X and others Y or nothing at all, they are actually contradictory. The extent to which distinct factors are deterministic in specific contexts is an important question in the present discussion, yet it is a separate empirical one. Only work that is specifically designed to tease out if and, if so, why AoA over proficiency over continuous bilingual engagement measures actually have distinct or potentially clandestine overlapping coverage. Trying to address this across the existent studies is inappropriate beyond speculation precisely because the requisite control is not in place. The issue should be acknowledged a priori, and resolving it should form part of future work.]

Notwithstanding predictions by the DRM and general models of experience-dependent neuroplasticity, the hypothesized inverted U-shaped neuroplastic association between hippocampal volume and L2 engagement has not yet been investigated in bilingual young adults. The present study fills this gap using structural MRI with an atlas-based morphometry approach.

## Materials and Methods

### Participants

Sixty-nine bilingual individuals (L1, Russian; L2, English; mean age, 22.81 years; SD, 3.4; 23 males) took part in the study (see Table 1 for an overview of demographic and language measures). All were right-handed, as indicated by the Edinburgh Handedness Inventory (Oldfield, 1971), and reported no psychiatric or neurological impairments. We assessed individual profiles for age, maximal educational attainment, and annual household income bands. The latter, used as a proxy of socioeconomic status, were adjusted based on the European Social Survey 2020 (ESS Round 10: European Social Survey Round 10 Data, 2020) to represent local standards. General intelligence was measured with a subset of the Raven's Standard Progressive Matrices for adults (Raven, 2003). The study was approved by the local research ethics committee, and written informed consent was obtained from all participants.

**Table 1.** Descriptive statistics for sociodemographic and language background measures

Variable	*N*	Mean	SD	Min	Max
L2 AOA (years old)	69	8.884	3.462	4	20
Engagement with L2 (daily %)	69	22.17	12.646	2	60
L2 Proficiency (Cambridge test score)	69	18.159	4.057	10	25
Years of education (years)	69	15.217	2.134	10	21
Age (years)	69	22.812	3.353	18	35
Socioeconomic status (7-level categorical)	69	4.942	1.748	2	7

### Experimental design

#### Bilingual experience

Participants were presented with the Russian version of the Language Experience and Proﬁciency Questionnaire (LEAP-Q; Marian et al., 2007) to evaluate their individual profiles along several dimensions of bilingual experience, including self-rated second language (L2) proficiency, L2 engagement, and AoA. To obtain an objective measure of L2 proficiency, participants also completed the online Cambridge test for adult learners (http://www.cambridgeenglish.org/test-your-english/general-english/).

#### MRI acquisition and preprocessing

T1-weighted images were acquired on a Philips Intera 1.5 T MRI scanner using the following parameters: TR, 25 ms; TE, 4.6 ms; flip angle, 30; FOV, 240 × 240; resolution, 1 × 1 × 1 mm; matrix, 256; TA, 5.35 min; mode, 3DFFE; and number of slices, 191. Bilateral hippocampal GMVs were extracted via a region-based morphometry routine implemented in CAT12 (Computational Anatomy Toolbox, https://neuro-jena.github.io/cat/) within the SPM12 (Statistical Parametric Mapping, https://www.fil.ion.ucl.ac.uk/spm/) software. Estimates of hippocampal volumes acquired with 1.5 T MRI scanners have been shown to be comparable with those acquired with 3 T scanners (Briellmann et al., 2001). Images were initially visually inspected to check for gross field distortions and movement artifacts, with no participants discarded as a result. The origin was then manually set to correspond to the anterior commissure–posterior commissure line. Subsequently, CAT12 segmentation procedure was used to segment raw structural images into GM, white matter (WM), and cerebrospinal fluid (CSF). CAT12 outputs postsegmentation reports providing automatized image quality ratings, which were checked to confirm that all images were of sufficient quality. Images were all rated substantially above the sufficiency level. In particular, the weighted image quality rating was A for 53 participants, A− for 15 participants, and B for 1 participant. We then coregistered each image to the International Consortium for Brain Mapping European brain space template with the affine regularization routine. After coregistration, bilateral hippocampal GMVs were estimated in non-normalized native space using maximum tissue probability labels from the Neuromorphometrics Atlas (http://www.neuromorphometrics.com/) via an in-built CAT12 tool. Total intracranial volume (TIV) was computed by summing global volumes of different tissue classes—GM, WM, and CSF—in native space. Finally, individual hippocampal GMVs were normalized against TIV following the procedure presented in Jack et al. (1989), to control for individual differences in the brain size.

### Statistical analysis

**Figure 1.**
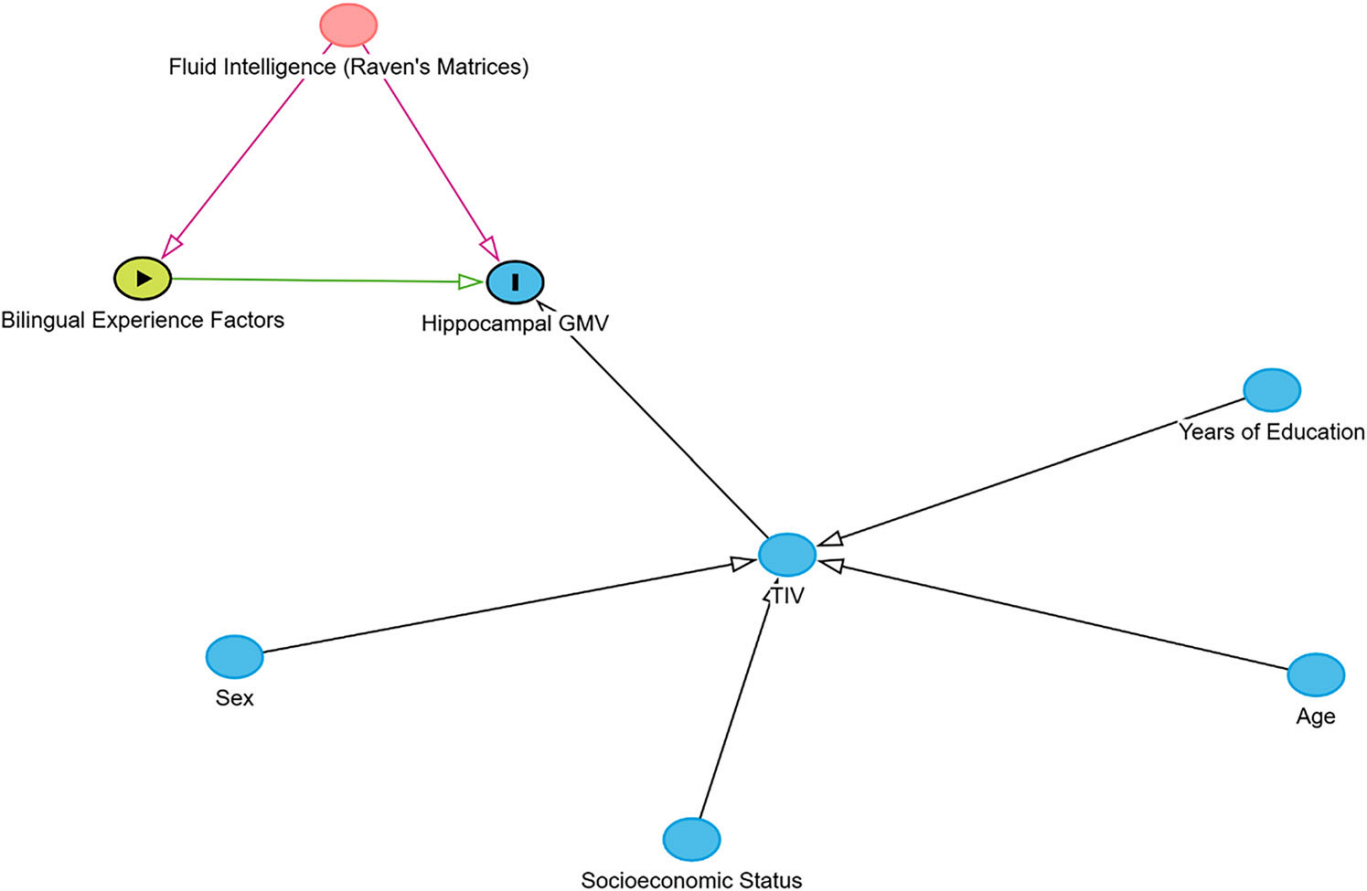
A causal diagram illustrating the reasons behind covariate structure choice for statistical models.

Statistical analyses were performed using Stata 17 (StataCorp, 2021). We used quadratic regressions to test whether variations in L2 proficiency, L2 AoA, and L2 engagement predicted variations in hippocampal GMVs. We evaluated which, among the sociodemographic measures we collected (i.e., fluid intelligence, years of education, sex, age, and socioeconomic status), to insert as covariates in our models. We expected fluid intelligence to affect both bilingual experience factors (BEFs) and hippocampal GMVs. Alternatively, we expected other variables, namely, sex, socioeconomic status, years of education, and age, to only influence hippocampal volumes and not BEFs. In particular, we expected these variables’ effect to be mediated by TIV, as there was no specific a priori motivation to expect them to influence hippocampal GMV particularly as opposed to whole-brain GMVs. The causal diagram illustrating our line of reasoning is visually represented in Figure 1. Since we had already adjusted our individual hippocampal volumes for TIV, and since these sociodemographic variables were not expected to influence our predictors of interest, i.e., BEFs, they were not included as covariates in the regression models to avoid noise inflation. Thus, our two full models—one with the left hippocampus GMV and the other with the right hippocampus GMV as the dependent variables—included L2 proficiency, L2 AoA, L2 engagement, and Raven's Matrices score as independent variables. To examine potential curvilinear relationships between BEFs and hippocampal GMVs, based on existing theoretical models (Pliatsikas, 2020), we interacted each BEF—specifically, L2 proficiency, L2 AoA, and L2 engagement—with itself in the regression models. This approach is used conventionally to allow the model to evaluate both the linear and quadratic contributions of each predictor simultaneously. The two quadratic regression models were tested via the *regress* function in Stata.

After model estimation, we ran a LASSO model selection procedure using a cross-validation selection method to identify the most parsimonious models, via Stata's *lasso linear* command.

## Results

For the right hippocampus, the full model (*R*^2^ = 0.156; *R*^2^_adjusted_ = 0.059) revealed no significant effect of BEFs, with Raven's Matrices score as the only significant predictor.

For the left hippocampus, the full model (*R*^2^ = 0.154; *R*^2^_adjusted_ = 0.057) revealed a significant effect of both the linear (*β* = 0.016; *p* = 0.035) and the curvilinear (*β* = −0.0003; *p* = 0.019) L2 engagement predictors, together with Raven's Matrices score (*β* = −0.148; *p* = 0.007). The curvilinear term for L2 engagement had a more significant contribution than the linear one, suggesting a curvilinear relationship between L2 engagement and GMV of the left hippocampus.

**Figure 2.**
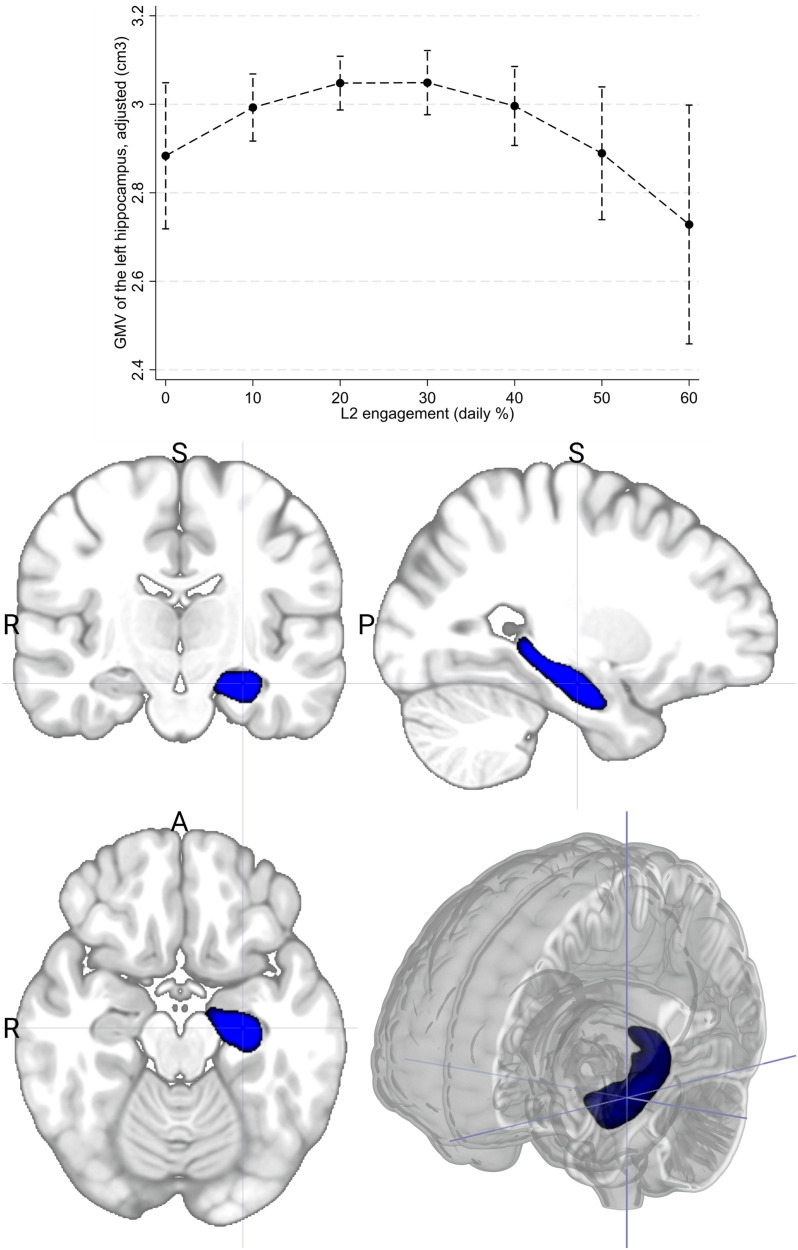
A plot of the curvilinear relationship between daily L2 engagement and left hippocampal GMV (top panel). The left hippocampus shown on the bottom panel on an MNI template for illustrative purposes.

For the right hippocampus, the LASSO procedure individuated as the best-fitting model (*R*^2^ = 0.083; *R*^2^_adjusted_ = 0.07) one including Raven's Matrices score as the sole predictor. For the left hippocampus, the best-fitting model (*R*^2^ = 0.139; *R*^2^_adjusted_ = 0.01) included Raven's Matrices Score and L2 engagement (linear, *β* = 0.014; *p* = 0.045; curvilinear, *β* = −0.0003; *p* = 0.025) as predictors. This confirmed the results of the full model analyses. After model estimation, we estimated the marginal effect of L2 engagement on left hippocampal GMV via Stata's *margins* command. The effect plot revealed that the relationship followed an inverted U-shape (Fig. 2).

## Discussion

Herein, we examined the association between individual differences in bilingual experience and variations in GMVs of the hippocampus. We hypothesized that this relationship would follow an inverted U-shaped pattern, consistent with the DRM and broader frameworks of experience-dependent neuroplasticity. Analyses confirmed our hypothesis: we observed an inverted U-shaped relationship between L2 engagement and GMV of the left hippocampus. This finding is in accord with theoretical models on the lifelong trajectory of bilingualism-related neuroplasticity. While we have introduced the DRM framework (Pliatsikas, 2020) above, it is worth unpacking it more explicitly at this juncture. Consider that the DRM predicts differential neural adaptations based on the time-course of bilingual experience and the relative variations in cognitive effort imposed by language learning and ensuing control demands. In other words, the DRM is predicated on the idea that the brain adapts continuously in response to bilingualism over time. The model proposes three stages—initial exposure (language learning), consolidation (development), and peak efficiency (maintenance after acquisition over time). Specifically for the hippocampus, the DRM predicts initial GMV increases followed by eventual renormalization (return to baseline) during the consolidation stage with potentially further reductions of GMV for those individuals who have reached peak efficiency. Crucially, however, the DRM also predicts measurably maintained or enhanced efficiency during the latter two stages, despite a structure that is renormalized or even reduced. As such, the hippocampus would be expected to increase in volume at initial stages of L2 acquisition in order to cope with cognitive demands associated with the novel task of language control. This novel cognitive demand would induce the hippocampus to undergo structural changes via the formation of new synaptic connections. Subsequently, increasing bilingual experience would lead to an increase of hippocampal functional efficiency. This increased functional efficiency, in turn, renders the previously accumulated “extra” structural resources no longer necessary for optimized language control. As consolidation of the learning process sets in, the surplus connections would thus be eliminated via synaptic pruning. This process would result in the hippocampal structural substrate to return to prebilingualism levels in gross volumetric terms (or even reduce), while its enhanced synaptic connectivity has been reorganized toward higher efficiency. Admittedly, the best way to capture this process would be in a longitudinal design. Nevertheless, the U-shaped pattern across our cross-sectional approach by relative exposure maps well onto these three hypothesized stages. It also replicates the pattern found in older bilingual populations with MCI (Voits et al., 2024) as well as data demonstrating similar volumetric trajectories in other cortical and subcortical regions of the language control/executive network (Marin-Marin et al., 2022; Gallo et al., 2023; Korenar et al., 2023; Yee et al., 2024). As such, we interpret this pattern as consistent with the DRM's predictions, highlighting what is at its core a nonlinear trajectory of economy-driven, experiential-based adaptation.

While we had not made any specific predictions regarding the lateralization of any effect, it must be noted that we only found a relationship between bilingual experience and the GMV of the left hippocampus. Indeed, previous literature in the neurocognition of bilingualism field has reported mixed findings regarding lateralization: bilingual experience has been reported to affect, similar to the present finding, the left hippocampus (Li et al., 2017) but also the right hippocampus (Mårtensson et al., 2012; Bellander et al., 2016) as well as both (DeLuca et al., 2019; Voits et al., 2022, 2024). Despite this, we would submit that the present lateralized pattern is not surprising. It is well known that hippocampal function is lateralized in healthy individuals, with the left hippocampus being dominant for linguistic cognitive performance (Nemati et al., 2023) and verbal memory (Ezzati et al., 2016). The left medial temporal lobe and the left hippocampus also seem to have a critical role in determining the hemispheric lateralization of language in general (Liégeois et al., 2004). With this in mind, it is not surprising that in the present sample of young healthy adults, the left hippocampus is specifically implicated. Indeed, in at least some of previous studies indicating either a bilateral or right lateralized hippocampus effect, it is not clear if this pattern is produced by dual language experience alone. For example, the very design of the Bellander et al. (2016) study involved high-intensity lexical learning. Accordingly, their findings might not reflect an effect of language experience per se but rather incipient, intense learning more generally. In the Mårtensson et al. (2012) study, the participants were simultaneous interpreters during rigorous army-based training which involved extensive lexicon memorizing routines; such an engagement in dual language use is clearly atypical in several ways, both in terms of an incomparable level of its intensity to everyday bilingual experiences (as in our study) and in how the brain is being taxed more generally. It is not clear that bilingual switching under such a scenario uses the same mechanisms, in part or entirely, as more mundane, yet typical bilingualism in more real-world contexts. As such, the studies mentioned above potentially capture elements taking place beyond (or in parallel to) the exponents of dual language engagement shared by the participants in the current study. Interestingly, in line with our results, Abutalebi et al. (2007) reported functional adaptation of the left hippocampus in bilinguals during literacy training. During an fMRI experiment, bilingual participants, who were only literate in their L2, learned to read words in their native language, which was reflected by significant activity of the left hippocampus and left caudate but only at the initial stages. At more proficient stages of the literacy acquisition process, these bilinguals would cease to rely on their left hippocampus.

As a final point, it is worth keeping in mind that substantial evidence points to bilingualism being a factor that contributes to better-than-expected longevity in the later years. Thus, bilingual experience is hypothesized to contribute to greater “reserve” (Perani and Abutalebi, 2015; Bialystok, 2021; Gallo et al., 2022)—a concept devised to account for the high variability observed in individual trajectories of cognitive aging. Reserve can be broadly defined as the individual capacity to resist adverse consequences of cognitive aging (Stern, 2009; Barulli and Stern, 2013; Stern et al., 2020). As such, it originates from cognitively challenging life experiences which reinforce one's neural and cognitive resources. In other words, reserve can be seen as the result of experience-dependent plasticity. Although the effects of reserve are mainly observable in senescence, its accrual is thought to take place over the course of the lifespan, starting from youth (Tucker and Stern, 2011). Early-life experiences appear to play a crucial role in shaping health and aging outcomes later in life (de Rooij, 2022), and higher general cognitive ability in youth is associated with greater reserve in older age, indicating that reserve-related compensatory mechanisms may depend, at least in part, on early-life experiences (Panico et al., 2023; Schwarz et al., 2024). In line with these findings, Lupien et al. (2007) have reported a relationship between early hippocampal volume and neurocognitive outcomes in late life stages. In this vein, our results provide an indication on the potential origin of the bilingualism-induced reserve effects extensively observed in the literature. Indeed, recall that both the DRM and the ESR predict, as a result of the inverted U-shaped neuroplastic trajectory, an increase in the efficiency of available neural resources related with the relevant cognitive ability. This augmented efficiency has been put forward as one of the mechanisms underlying the protective effects exerted by reserve during the aging process (Barulli and Stern, 2013). Further supporting this interpretation is the observation that the present results parallel previous findings of a curvilinear relationship between bilingual experience and hippocampal volumes in older adults with MCI (Voits et al., 2024).

In summary, the present neuroimaging findings add to the body of evidence showing that cognitive demands associated with dual language acquisition and control lead to neurostructural adaptations in the brain, documented here for the hippocampus—a brain structure critical for a vast array of cognitive functions. The present study underscores the role of bilingualism as a powerful promoter of experience-dependent neuroplasticity.

## Synthesis

Reviewing Editor: James Howard, Brandeis University Decisions are customarily a result of the Reviewing Editor and the peer reviewers coming together and discussing their recommendations until a consensus is reached. When revisions are invited, a fact-based synthesis statement explaining their decision and outlining what is needed to prepare a revision will be listed below. The following reviewer(s) agreed to reveal their identity: John Anderson. Note: If this manuscript was transferred from JNeurosci and a decision was made to accept the manuscript without peer review, a brief statement to this effect will instead be what is listed below.

This manuscript examines nonlinear contributions to hippocampal gray matter volume in aging. The paper expands work on the role of hippocampal plasticity in bilingualism and effects related to experience-dependent neuroplasticity. The paper is well-written, with carefully conducted analyses, though the cross-sectional nature of the study remains a limitation. This is a revised manuscript and the authors have addressed all the points raised and made significant changes to the manuscript in response to the reviewers' feedback. We particularly appreciate the care the authors took in revising the introduction and the statistics - including the directed acyclic graph as a justification for inclusion of necessary covariates was a nice touch.
